# Assessing survival in widowers, and controls -A nationwide, six- to nine-year follow-up

**DOI:** 10.1186/1471-2458-12-96

**Published:** 2012-02-02

**Authors:** Bragi Skulason, Lilja Sigrun Jonsdottir, Valgerdur Sigurdardottir, Asgeir R Helgason

**Affiliations:** 1 University of Iceland, Saemundargata, Reykjavik IS101, Iceland; 2 National University Hospital, Eiriksgata 29, Reykjavik IS101, Iceland; 3 Reykjavík University, Menntavegur 1, Reykjavik IS101, Iceland; 4 Directorate of Health, Austurstrond 5, Seltjarnarnes IS170, Iceland; 5 National University Hospital, Palliative Care Unit, Kopavogur IS200, Iceland; 6 Department of Public Health Sciences, Social Medicine, Karolinska Institutet, Stockholm SE17177, Sweden

## Abstract

**Background:**

The aim of this study was to assess if widowers had an increased mortality rate during the first 6 to 9 years after the death of their wife, compared initially to an age-matched control group and also compared to the general population of Iceland.

**Methods:**

The study base was comprised of all 371 men born in 1924-1969 who were widowed in Iceland in 1999-2001 and 357 controls, married men, who were matched by age and residence.

The widowers and controls were followed through the years 2002-2007 using information from Statistics Iceland. Mortality rates were compared between the groups and also with the general population. The mortality rate comparisons were: study group vs. control group, on the one hand, and study group vs. general population on the other. Causes of death were also compared between widowers and their wives.

**Results:**

A statistically significant increase in mortality in the widowers' group, compared to controls, was observed.

Lifestyle-related factors could not be excluded as contributing to cause of death in these cases.

**Conclusions:**

Being a widower was related to an increased risk of death for at least 9 years after the death of their wife.

## Background

Studies have shown that widowers seem to have a higher risk of mortality after spousal death compared to other males [1-13]. Some previous findings indicated an early excess risk of mortality (e.g. in the first six months) [5,14-16], but others have noted increased risk persisting for a longer period of time [6,17,18], and for selected causes of death [19]. One study showed no strong statistical evidence that the increased risk of death associated with bereavement changed with time after bereavement [20]. Compared to widows, widowers seem to have a higher risk of morbidity and mortality [5,15,21-27].

For more than a century epidemiologists have studied the influence of marital status on mortality [28]. Most of these studies have been performed in selected or small populations with conflicting results [29]. A recent large epidemiological study from the US indicated an 18% increased risk of death for widowers compared to non-widowers over a period of 9 years [30]. However, there is a scarcity of studies with samples representative of a whole population in which widowers are followed up over a longer period of time.

In addition, theories explaining observed increased mortality rates in widowers after loss of spouse are just emerging, and empirical data are also limited. A Swedish study from the 1980's concluded that lifestyle factors could not be excluded as a possible explanation for excess mortality among widowers and widows [29].

Although data are scarce a large body of evidence from the scientific literature strongly links some behavioral risk factors to both cancer and cardiovascular disease groups. These groups most frequently include smoking, excessive alcohol use, dietary factors, physical activity, and overweight and obesity [21,31].

The primary aim of this study was to assess whether the widowers had an increased mortality rate during the first 6 to 9 years after the death of their wife as compared to the death rates in an age-matched control group; and secondly, to compare widowers' mortality to the general population. Additionally, the authors investigated whether there may be a pattern in causes of death of married couples as reflected in registered causes of death.

The present study is the first nationwide study of widowers' mortality in Iceland. It is an extension of a study on widowers assessing widowers' psychological and physiological well-being and comparing them with an age-matched control group of married men over a period of 6 to 9 years [32].

## Methods

A general census of the Icelandic population takes place on December 1st every year and is conducted by Statistics Iceland. On December 1, 2001 the total population of Iceland was 286,575 people, of which 143,450 were men and 143,125 were women [33].

All individuals in Iceland have a unique state personal identification number issued at birth. When the information for this study was gathered the personal identification number was used to find the study population.

The study population was identified on December 31st, 2001, with the primary aim of comparing widowers' bereavement with a representative age-matched control group, assessing psychological and physiological well-being. However, during the data collection process the research group decided to add a mortality assessment analysis as a secondary aim.

### Widowers

The original study base of widowers included all 357 Icelandic widowers born in 1924-1969 who had lost their wives during the years 1999-2001 and were alive and living in Iceland on December 31st, 2001 (Table 1). At the time of selection, 14 widowers had already died but they were included in the survival analysis comparing widowers with the general population (Table 2).

**Table 1:** Baseline (demographic) information on widowers who lost their wife during 1999-2001, and a control group of age-matched married men.

	Widowers	Controls
**All widowers who lost their wives 1999-2001, born 1924-1969, and age-matched controls**	N = 357	N = 357

Mean age at time of selection	63,9 (30-75)	62,5 (35-75)

Age group 30-59	n = 107	n = 107

Age group 60-69	n = 126	n = 126

Age group 70-75	n = 124	n = 124

Participants of epidemiological study*	N = 216 % (n/N)	N = 199 % (n/N)

Years married 20-40 years	37% (80/216)	43% (86/199)

Years married 40+ years	51% (110/216)	47% (94/199)

Living in Reykjavík metropolitan area	61% (131/216)	63% (126/199)

Living in towns outside of Reykjavík	31% (66/216)	28% (55/199)

Living in a rural area	8% (18/216)	9% (18/199)

Have children-all ages	97% (208/216)	98% (195/199)

Education after age 16		

0-2 years	34% (74/216)	30% (60/199)

3-4 years	37% (79/216)	40% (79/199)

5-10 years	22% (48/216)	28% (56/199)

missing	7% (15/216)	2% (4/199)

Work status		

Retired	38% (81/216)	36% (71/199)

Carpenter	12% (25/216)	20% (40/199)

Office job	12% (25/216)	10% (19/199)

Own company/director	9% (19/216)	11% (21/199)

Manual labourer	5% (10/216)	6% (12/199)

Merchant	6% (13/216)	8% (15/199)

Teacher	4% (9/216)	5% (9/199)

Pastor	2% (5/216)	0% (0/199)

Farmer	2% (4/216)	3% (5/199)

Fisherman	1% (3/216)	4% (7/199)

Other/missing	10% (22/216)	0% (0/199)

Information from a questionnaire survey in 2002

* Detailed information is only available from the participants of the primary epidemiological study

* Log-rank test *p *= 0.0121

**Table 2:** Relative survival and 95% CI of widowers (n = 371) and general population Jan 1^st ^1999-Jan 1^st ^2008.

Years after death of spouse	Number of widowers alive at the beginning of the interval	Crude survival	Relative survival	Lower 95% CI for relative survival	Upper 95% CI for relative survival
0-1	371	99.5%	> 100%	100%	> 100%
1-2	369	95.7%	99.1%	96%	> 100%
3-4	355	93.3%	98.5%	95%	> 100%
4-5	346	91.4%	98.6%	95%	> 100%
5-6	339	88.9%	98.2%	94%	> 100%
6-7	330	86.8%	98.3%	94%	> 100%

Crude survival and number at risk in the beginning of time interval is also shown. The Finnish program SURV3 (Windows Software for Relative Survival) was used for calculating relative survival

No statistical significance

For reasons of clarification, only widowers who had been legally married were included in the study base, and the same was true for the controls.

### Wives

In the original study base there were 357 wives born in 1916-1969 who had died; including the wives of the 14 widowers who had already died at the time of selection the total number of wives was therefore 371. Only 13 wives of the controls died in 2002-2007 (not in table).

### Controls

On December 31st, 2001, a control group comprised of 357 married men was randomly selected from the data from Statistics Iceland. The controls were matched with the widowers according to age and place of residency (Table 1).

### Statistical analysis

Relative survival is defined as the ratio of the observed survival in the group of widowers compared to the survival expected in a group of people in the general population (Table 2) who are similar to the widowers with respect to sex, age and calendar time at the time. It can be interpreted as the probability of the widowers' survival in the absence of other causes of death. The Finnish program SURV3 (Windows Software for Relative Survival) [34] was used for calculating relative survival (Table 2).

Widowers whose wives died in 1999, 2000 and 2001 and were still living on December 31st, 2001, were followed by mortality rate to December 31st, 2007. The crude and relative survival rates between the widowers and men in the general population, corrected by birth year, was carried out (Table 2), using mortality statistics from Statistics Iceland [33], and the relative risk (95% CI) was calculated.

The Kaplan-Meier method [35] was used for the univariate analysis of survival, and the Log Rank test used for estimating equality of survivor functions. Relative hazards were estimated using the Cox proportional hazards model (corrected for birth year) [36].

The analyses were performed using STATA Statistical Software Stata 10 for Windows [37].

Detailed baseline (demographic) information was available on the participants from our initial epidemiological study (N = 357) and controls (N = 357). It is presented in Table 1. Baseline information on the 14 widowers who had died during 1999-2001 was not available.

### Time and cause of death

Time and causes of death are registered in Statistics Iceland using the state personal identification numbers. All causes of death are registered based on WHO's ICD-10 system (International Classification of Diseases) [38]. Time and cause of death of the widowers' wives was identified at the start of the study. Throughout the study period, time and cause of death was continuously registered for the widowers as well as for the controls and their wives. We classified causes of death into three main categories, including cardiovascular disease, cancer, and other causes, and divided the population by similar size age groups 30-59, 60-69 and 70-75 years old for the men, and 30-59, 60-69 and 70+ (some of the wives of widowers were older than 75) for the women (not in table).

### Lifestyle risk factors

All causes of death were evaluated regarding following risk factors: smoking, excessive alcohol use, dietary factors, physical activity, overweight and obesity.

### Ethical aspects

This study was approved by the National Bioethics Committee of Iceland, number VSN 200620033/03-7, the Icelandic Data Protection Authority, number 2006020102, and the Icelandic National Registry. Confidentiality and anonymity were of utmost importance. Approval was sought for expansion of the ongoing epidemiological study assessing widowers' psychological and physiological well-being to include a follow-up of the men to assess differences in mortality rates up to 9 years post-spousal loss. The original epidemiological study was anonymous. Thus, no direct assessment could be made to analyze the relation of well-being to mortality.

## Results

### Baseline (demographic) information

Approximately 50% of the widowers and controls had lived in wedlock for 40 years or more and 97% of them had children (Table 1). The employment distribution in both groups was representative of the Icelandic population. Over 60% of the widowers and controls lived in the Reykjavik metropolitan area. Approximately 30% of the widowers lived in other townships around Iceland and about 10% lived in a rural setting. These numbers correspond well with the distribution of the general population, available from Statistics Iceland.

### Survival estimates

**Figure 1:**
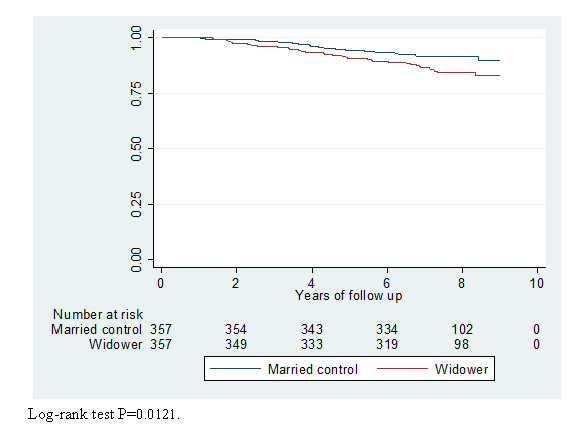
**Kaplan -Meier survival estimates**. Group of age-matched widowers and a control group of married men. The group is followed from Jan 1st 1999 to Jan 1st 2008.

Survival estimates adjusted for year of birth were calculated comparing widowers with controls (Table 3 and Figure 1). As observed in Table 3 the differences in survival estimates were significant between the groups from the second year after loss and the differences in survival appeared to increase over time. The differences in survival between the groups over time are illustrated using the Kaplan-Meier survival estimates presented in Figure 1, and the log-rank test for equality of survivor function (*p *= 0.0003) (Figure 1).

**Table 3:** Kaplan-Meier survival estimates.

year after	controls		widowers	
losing spouse	survival	95% CI	survival	95% CI
1	100		100	
2	99.2	97.4-99.7	97.8	95.6-98.9
3	98.3	96.3-99.2	96.4	93.8-97.9
4	96.1	93.5-97.7	93.3	91.1-95.4
5	94.4	91.5-96.4	91.0	87.6-93.6*
6	93.6	90.5-95.7	89.4	85.7-92.1
7	91.6	88.2-94.1	86.5	82.3-89.8
8	91.6	88.2-94.1	84.6	80.0-88.2*

Age matched widowers (n = 357) versus controls (n = 357)

*Statistical significance between widowers and controls

Cox regression's hazard ratio with a 95% confidence interval of 1.84 (1.19-2.86) was corrected by birth year, i.e. widowers are 1.84 times more likely to be at risk than the controls. This difference was statistically significant (Figure 1).

### Comparison with the general population

An additional crude and relative survival analysis was done comparing widowers with the general population of all Icelandic men born in 1924-1969 revealing a crude survival rate, independent of cause of death, of 86.8% 6 years after spousal death (Table 2) and a 6 year relative survival of 98.3% (Table 2).

### Causes of death

Since all causes of death were identifiable, we include Table 4, which shows causes of death divided into three categories: cardiovascular disease, cancer, and other causes.

**Table 4:** A comparison of causes of death from cardiovascular diseases, cancer and other causes during the years 1999-2007.

	Controls: Deaths 2002-2007 n = 30			Widowers: Deaths 1999-2007 n = 64		
	**Age 30-59 years-old n = 2**	**Age 60-69 years-old n = 11**	**Age 70-75 years-old** **n = 17**	**Age 30-59 years-old n = 4**	**Age 60-69 years-old n = 18**	**Age 70-75 years-old** **n = 42**

Cardiovascular disease - % (n)	0% (0)	36% (4)	18% (3)	75% (3)	33% (6)	26% (11)

Cancer - % (n)	50% (1)	45% (5)	65% (11)	0% (0)	39% (7)	48% (20)

Other - % (n)	50% (1)	18% (2)	18% (3)	25% (1)	28% (5)	26% (11)

Comparing Icelandic widowers (born 1924-1969) and the controls

Total population of men 1999, n = 138,086 Info obtained from Statistics Iceland

At the end of the follow-up period (January 1 2008) 64/371 (17.3%) of the widowers had died (Table 4). In the control group 8% (30/357) of the men had died (Table 4) and 4% (13/357) of the wives (not in table). However, there were no cases in the control group where both spouses had died during the time of the study.

Diseases of the respiratory system, external causes of injury and poisoning, as well as other causes of death, accounted for a similar proportion for both widowers and their wives, and also for the controls (not in table).

A comparison between the leading causes of death (cancer and cardiovascular diseases) was difficult since there were few wives who had died in the control group. However, as expected, the ratio of cardiovascular diseases and cancer versus other causes of death was significantly higher (91%) in the oldest group of women compared with the younger groups (not in table). When comparing widowers and controls for cancer and cardiovascular diseases, however, except for the youngest group, where there were very few deaths, the older groups of both widowers and controls were above 70% (Table 4).

Of the 64 deceased couples, 80% (51/64) of the deceased wives had died of either cancer or cardiovascular diseases (not in table). For the widowers the proportion was 73% (47/64).

In 63% (40/64) of the cases, both couples died of cancer and/or cardiovascular diseases. After questionnaires were sent out in 2003, 1-3.5 years after the widowers had lost their wives, only 7 widowers were registered as remarried or living with another woman (not in table).

## Discussion

In this study, widowers were shown to have a significantly lower survival rate compared with an age-matched control group and the difference in mortality rate increased over the period of the study period. The lower survival rate for the widowers was also apparent when comparing them to the general population of age-matched men. Common lifestyle risk factors could not be excluded as a contributing factor for the differences in mortality risk.

### Study setting

The study documents a survival analysis of all Icelandic widowers aged 30-75 years at the time of their wife's death. The widowers were followed up for 6 to 9 years. The mortality risk in the widowers' group was compared to that of randomly selected, age-matched controls, as well as to the mortality risk in the general population.

The present study was an extension of an epidemiological nation-wide study assessing widowers' psychological and physiological well-being and comparing them to a group of age-matched married men. Parts of that study have been previously published [32].

It soon became apparent during the follow-up period of the original study group that the number of widowers who died prior to follow-up exceeded the death rate in the control group. Thus, this study was designed, and a decision was made to follow the groups over time.

### Baseline (demographic) information

The baseline information presented in Table 1 was only available from the responding participants in the primary epidemiological study, comprised of 61% (216) of the widowers and 56% (199) of the controls.

Comparing the demographic data between the groups showed that the groups were indeed very similar (Table 1). There was no indication that the 14 widowers (baseline information not available) who had died before the time of selection of the control group were significantly different from the rest of the widowers regarding the assessed demographic variables or that including them would compromise the comparability between the widowers and controls.

### Comparing survival estimates

In the comparison between the control group and the widowers presented in Table 3, the 14 widowers who had died before the selection date for the control group were included. Information from Table 3 shows an increased mortality in the widowers' group and that it was statistically significant compared to that of the control group.

In Table 2, we compare survival in the general population of age-matched men with survival in the widowers' group, including the 14 widowers who had died during the 3-year period (1999-2001). The differences in crude survival estimates increased over the years (Table 2). The relative survival of widowers and the general population also showed a progressive increase in mortality in the widowers' group (Table 2). However, as expected the 95% confidence interval in the relative survival analysis for this comparison was only bordering on statistical significance (Table 2). There were several problems with comparing the widowers with all aged-matched men from the general population (Table 2). The general population also included the widowers. In a country with a small population this may have some (although small) effect on the comparison. More importantly, the general population data was comprised of all men, including those who had been chronically ill for a long time.

The effect of remarriage (or living with another woman) among widowers (2%) or the widowed among the controls (4%) most probably did not affect the results since there were very few of these cases (not in table).

### Other studies on widowhood mortality

Increased probability of death amongst widows and widowers has been found in bereaved men and women of all ages around the world, using cross-sectional and longitudinal data, with and without covariate controls, and diverse statistical methodologies [9,11,39-41]. Some studies have found an elevated mortality risk for bereaved individuals but no effects of covariates on the association between widowhood and mortality, which indicates that bereavement does not necessarily have a causal effect on survival [16,18]. An empirical study of spousal bereavement in southern Sweden revealed that widowers in general were found to have a higher relative mortality than widows. In that study, the effects of bereavement decreased over time [40].

There is limited data in the literature addressing why widowed people have a higher mortality risk compared with other age- and gender-matched groups [3]. Many potential risk factors are still under-researched [2]. Two recent epidemiological studies assessing increased mortality after spousal death reported strong indications that lifestyle-related diseases may, to a large extent, explain the observed increased mortality in the wives [3,29]. The effects of bereavement on mortality was investigated in all widowed people in Sweden from 1968 to 1978, showing a significant increase in widowers' mortality during the first 3 months after bereavement (up by 48%) and excess mortality at a lower level for 11 years following spousal loss. Lifestyle factors could not be excluded as a possible explanation for excess mortality among widowers and widows [29]. A Spanish study covered all individuals 25 years and older who died in Spain in 1991. The mortality risk was in all cases higher in single and widowed persons compared to married persons [42]. From Denmark, a comparative study of losing a spouse and losing a co-twin showed a greater relative increase in mortality amongst the widowers during the first year. However, after the first year, the surviving twins' mortality risk became higher than that of the widowers [43]. A prospective study of mortality from Finland reported an excessive mortality risk for both widows and widowers, but the relative mortality risk was higher for the widowers. They concluded that distress due to bereavement may have been directly related to increased mortality during the first months of bereavement, but that this effect appeared to be relatively small [6]. Another Finnish epidemiological study reported an elevated relative mortality risk immediately after bereavement [4].

Overall, it appears that studies on mortality in widowed people show an increased risk of mortality. Widowers appear to have a relatively higher mortality risk than widows. There may be a distress-related factor explaining some of this increase in mortality risk, but that appears to be a short-term effect. Shared lifestyle risk factors have been suggested as a possible explanation for long-term increased mortality in widowhood.

### Causes of death

Several studies have shown that people living together over a long period of time tend to have similar health risk behaviors [11,39-41,43-45]. Most cancers and cardiovascular diseases are strongly related to lifestyle risk factors such as smoking, alcohol use, diet, lack of physical activity, overweight and obesity [19-21,24,46].

Although we did not assess lifestyle in the present study, we were able to identify all causes of death in all subjects, according to WHO's International Classification of Diseases. A conservative approach was chosen to compare causes of death in 3 groups, cancer, cardiovascular diseases and other causes (Table 4). There was an overrepresentation of cancer deaths for widowers' wives in all age groups (not in table), but there were very few deceased wives in the control group (13) and, therefore, comparison was difficult. Cancer deaths follow a similar pattern between all age groups when comparing widowers and controls (Table 4), and the same is true for cardiovascular diseases.

Probably the most interesting finding from the cause-of-death analysis was the fact that in the control group there were no cases where both couples had died during the follow-up period, in contrast to 17.3% (64/371) of couples in the widowers' group.

The main strength of our study is that it was based on a total national sample of widowers, comprised of all widowers up to the age of 75 during a 3-year period. In addition, we were able to compare the mortality risks, both with a control group of married men as well as population-based statistics for all men in Iceland during a 6-to-9 year follow-up. Both approaches have limitations and strengths. Unfortunately, we were unable to relate causes of death to particular lifestyle factors due to the study design, since lifestyle assessment was not a part of the original study.

An important weakness of the present study was that it was not a mortality study from the start. Also, due to anonymity the information obtained in the questionnaire could not be used to analyze risk factors.

### Public health implications

Assessing and describing the health consequences of widowhood is an understudied area of public health research. Understanding how widowhood may impact health and survival may help us to better understand how to support people in these situations.

## Conclusion

Becoming a widower is related to an increased risk of death for at least 6 to 9 years after the death of the spouse. A progressive increase of mortality in the widowers' group was statistically significant compared to controls. This trend continued throughout the study period. Increased morbidity from shared lifestyle risk factors could not be excluded as an explanation for the observed mortality rates in the widowers.

## Competing interests

The authors declare that they have no competing interests.

## Authors' contributions

ARH supervised the study design, analysis and writing of the paper. BS identified all subjects in the study base, worked on the analysis and the writing of the paper, and collaborated with the Icelandic National Registry (Statistics Iceland). VS contributed with the writing of the introduction and discussion as well as in the assessment of the possible relation between health-risk related lifestyle and cause of death. LSJ contributed in the mortality analyses and the assessment of the possible relation between health-risk related lifestyle and cause of death. The interpretation of data was done jointly by ARH, BS, VS and LSJ. All authors have read and approved the final manuscript.

## Pre-publication history

The pre-publication history for this paper can be accessed here:

http://www.biomedcentral.com/1471-2458/12/96/prepub

## References

[B1] Stroebe MS Stroebe W Schut H Gender differences in adjustment to bereavement: an empirical and theoretical review Rev Gen Psychol 2001 5 62 83

[B2] Stroebe MS Schut H Stroebe W Health outcomes of bereavement Lancet 2007 370 1960 1973 10.1016/S0140-6736(07)61816-9 18068517

[B3] Elwert F Christakis NA The effect of widowhood on mortality by the causes of death of both spouses Am J Public Health 2008 98 2092 2098 10.2105/AJPH.2007.114348 18511733 PMC2636447

[B4] Kaprio J Koskenvuo M Rita H Mortality after bereavement: A prospective study of 95,647 widowed persons Am J Public Health 1987 77 283 287 10.2105/AJPH.77.3.283 3812831 PMC1646890

[B5] Lichtenstein P Gatz M Berg S A twin study of mortality after spousal bereavement Psychol Med 1998 28 635 643 10.1017/S0033291798006692 9626719

[B6] Martikainen P Valkonen T Mortality after death of spouse in relation to duration of bereavement in Finland J of Epidemiol & Comm Health 1996 50 264 268 10.1136/jech.50.3.264 8935456 PMC1060281

[B7] Brockmann H Klein T Love and death in Germany: The marital biography and its effect on mortality J of Marriage and Family 2004 66 3

[B8] Hemström Ö Is marriage dissolution linked to differences in mortality risks for men and women? J of Marriage and the Family 1996 58 366 378 10.2307/353502

[B9] Hu Y Goldman N Mortality differentials by marital status: An international comparison Demography 1990 27 233 250 10.2307/2061451 2332088

[B10] Stimpson JP Kuo YF Ray LA Raji MA Peek MK Risk of mortality related to widowhood in older Mexican Americans Ann Epidemiol 2007 17 313 319 10.1016/j.annepidem.2006.10.006 17306987 PMC2700854

[B11] Helsing KJ Comstock GW Szklo M Causes of death in a widowed population Am J of Epidemiology 1982 116 3 524 532 10.1093/oxfordjournals.aje.a113436 7124718

[B12] Johnson NJ Backlund E Sorlie PD Loveless CA Marital status and mortality: The National Longitudinal Mortality Study Ann Epidemiol 2000 10 224 238 10.1016/S1047-2797(99)00052-6 10854957

[B13] Mineau GP Smith KR Bean LL Historical trends of survival among widows and widowers Social Sci Med 2002 54 245 254 10.1016/S0277-9536(01)00024-7 11824929

[B14] Manor O Eisenbach Z Mortality after spousal loss: Are there socio-demographic differences? Social Sci Med 2003 56 405 413 10.1016/S0277-9536(02)00046-1 12473324

[B15] Martikainen P Valkonen T Mortality after the death of a spouse: Rates and causes of death in a large Finnish cohort Am J of Pub Health 1996 86 1087 1093 10.2105/AJPH.86.8_Pt_1.1087 PMC1380614 8712266

[B16] Mendes de Leon CF Kasl SV Jacobs S Widowhood and mortality risk in a community sample of the elderly: a prospective study J Clin Epidem 1993 46 519 527 10.1016/0895-4356(93)90124-J 8501478

[B17] Li J Precht DH Mortensen PB Olsen J Mortality in parents after death of a child in Denmark: a nationwide follow-up study Lancet 2003 361 363 367 10.1016/S0140-6736(03)12387-2 12573371

[B18] Schaefer C Quesenberry CP Jr Wi S Mortality following conjugal bereavement and the effects of a shared environment Am J Epidemiol 1995 141 1142 2252 7771452 10.1093/oxfordjournals.aje.a117387

[B19] West of Scotland Coronary Prevention Group Baseline risk factors and their association with outcome in the West of Scotland Coronary Prevention Study Am J Cardiol 1997 79 6 756 762 10.1016/S0002-9149(96)00863-6 9070554

[B20] Hart CL Hole DJ Lawlor DA Smith GD Lever TF Effect of conjugal bereavement on the mortality of the bereaved spouse in participants of the Renfrew/Paisley Study J Epidemiol Comm Health 2007 61 455 460 10.1136/jech.2006.052043 PMC2465697 17435215

[B21] Hemminki K Li X Lifestyle and cancer: Effect of widowhood and divorce Cancer Epidemiol, Biom & Prevention 2003 12 899 904 14504201

[B22] Stimpson JP Kuo YF Ray LA Raji MA Peek MK Risk of mortality related to widowhood in older Mexican Americans Ann Epidemiol 2007 17 313 319 10.1016/j.annepidem.2006.10.006 17306987 PMC2700854

[B23] Stroebe MS Stroebe W Who suffers more? Sex differences in health risks of the widowed Psychol Bull 1983 93 279 301 6844474

[B24] Umberson D Wortman CB Kessler RC Widowhood and depression: Explaining long-term gender differences in vulnerability J Health Soc Behav 1992 33 10 24 10.2307/2136854 1619255

[B25] Lee GR DeMaris A Bavin S Sullivan R Gender differences in the depressive effect of widowhood in later life J Gerontol B Psychol Sci Soc Sci 2001 56 S56 S61 10.1093/geronb/56.1.S56 11192346

[B26] van Grootheest DS Beekman AT Broese van Groenou MI Deeg DJ Sex differences in depression after widowhood: Do men suffer more? Soc Psychiatry Psychiatr Epidemiol 1999 34 391 398 10.1007/s001270050160 10477960

[B27] Li G The interaction effect of bereavement and sex on the risk of suicide in the elderly: a historical cohort study Soc Sci Med 1995 40 825 828 10.1016/0277-9536(94)00135-G 7747217

[B28] Susser M Widowhood: A situational stress or a stressful life event Am J of Pub Health 1981 71 793 795 10.2105/AJPH.71.8.793 PMC1619996 7258439

[B29] Mellström D Nilsson Å Odén A Rundgren Å Svanborg A Mortality among the widowed in Sweden Scand J Soc Med 1982 10 33 41 7178869 10.1177/140349488201000201

[B30] Subramanian SV Elwert F Christakis N Widowhood and mortality among the elderly: the modifying role of neighborhood concentration of widowed individuals Soc Sci Med 2008 66 4 873 884 10.1016/j.socscimed.2007.11.029 18178300 PMC2790530

[B31] Martin-Moreno JM Soerjomataram I Magnusson G Cancer causes and prevention: a condensed appraisal in Europe in 2008 Eur J Cancer 2008 44 10 1390 1403 10.1016/j.ejca.2008.02.002 18329264

[B32] Skulason B Helgason AR Identifying obstacles to participation in a questionnaire survey on widowers' grief BMC Palliative Care 2010 9 7 10.1186/1472-684X-9-7 20429883 PMC2873496

[B33] Statistics Iceland (The centre for official statistics in Iceland). Population Unit Databases 2010

[B34] SURV3 http://www.cancer.fi/@Bin/54321472/index.html

[B35] Kaplan EL Meier P Nonparametric estimation from incomplete observations J Am Stat Assoc 1958 53 457 481 10.2307/2281868

[B36] Cox proportional hazard model 2006 http://userwww.service.emory.edu/~poldd/survival3.pdf

[B37] Dickman P Coviello E Hills M Estimating and modelling relative survival using Stata 2007 http://www.pauldickman.com/rsmodel/stata_colon/ATH laga línubil

[B38] WHO's International Classification of Diseases 2010 http://www.who.int/classifications/icd/en/

[B39] Lillard LA Waite LJ Till death do us part: Marital disruption and mortality Am J Soc 1995 100 1131 1156 10.1086/230634

[B40] Nystedt P Engelen T, Kok J Widowhood-related mortality in Scania, Sweden during the 19th century The History of the Family 7 2002 3 451 478 10.1016/s1081-602x(02)00113-6 20707030

[B41] Parkes CM Benjamin B Fitzgerald RG Broken Heart: a statistical study of increased mortality among widowers BMJ 1969 1 5646 740 743 10.1136/bmj.1.5646.740 5769860 PMC1982801

[B42] Burgoa M Regidor E Rodriguez C Gutierrez-Fisac JL Mortality by cause of death and marital status in Spain Eur J Pub H 1998 8 37 42 10.1093/eurpub/8.1.37

[B43] Tomassini C Rosina A Billari FC Skytthe A Christiansen K The effect of losing the twin and losing the partner on mortality Twin Res 2002 5 210 217 10.1375/136905202320227871 12184889

[B44] Klein T Soziale Determinanten der Lebenserwartung (Social determinants of life expectancy) Kölner Zeitschrift für Soziologie und Sozialpsychologie 1993 45 712 730 12289802

[B45] Kraus AS Lilienfeld AM Some epidemiologic aspects of the high mortality rate in the young widowed group J Chr Dis 1959 10 207 217 10.1016/0021-9681(59)90003-7 14411769

[B46] Ford ES Critchley JA Labarthe DR Kottke TE Giles WH Capewell S Explaining the decrease in U.S. deaths from coronary disease, 1980-2000 N Engl J Med 2007 356 23 2388 2398 10.1056/NEJMsa053935 17554120

